# PCAT-UNet: UNet-like network fused convolution and transformer for retinal vessel segmentation

**DOI:** 10.1371/journal.pone.0262689

**Published:** 2022-01-24

**Authors:** Danny Chen, Wenzhong Yang, Liejun Wang, Sixiang Tan, Jiangzhaung Lin, Wenxiu Bu

**Affiliations:** 1 College of Information Science and Engineering, Xinjiang University, Urumqi, Xinjiang, China; 2 Key Laboratory of Multilingual Information Technology in Xinjiang Uygur Autonomous Region, Xinjiang University, Urumqi, Xinjiang, China; University of Engineering & Technology, Taxila, PAKISTAN

## Abstract

The accurate segmentation of retinal vessels images can not only be used to evaluate and monitor various ophthalmic diseases, but also timely reflect systemic diseases such as diabetes and blood diseases. Therefore, the study on segmentation of retinal vessels images is of great significance for the diagnosis of visually threatening diseases. In recent years, especially the convolutional neural networks (CNN) based on UNet and its variant have been widely used in various medical image tasks. However, although CNN has achieved excellent performance, it cannot learn global and long-distance semantic information interaction well due to the local computing characteristics of convolution operation, which limits the development of medical image segmentation tasks. Transformer, currently popular in computer vision, has global computing features, but due to the lack of low-level details, local feature information extraction is insufficient. In this paper, we propose Patches Convolution Attention based Transformer UNet (PCAT-UNet), which is a U-shaped network based on Transformer with a Convolution branch. We use skip connection to fuse the deep and shallow features of both sides. By taking advantage of the complementary advantages of both sides, we can effectively capture the global dependence relationship and the details of the underlying feature space, thus improving the current problems of insufficient extraction of retinal micro vessels feature information and low sensitivity caused by easily predicting of pixels as background. In addition, our method enables end-to-end training and rapid inference. Finally, three publicly available retinal vessels datasets (DRIVE, STARE and CHASE_DB1) were used to evaluate PCAT-UNet. The experimental results show that the proposed PCAT-UNET method achieves good retinal vessel segmentation performance on these three datasets, and is superior to other architectures in terms of AUC, Accuracy and Sensitivity performance indicators. AUC reached 0.9872, 0.9953 and 0.9925, Accuracy reached 0.9622, 0.9796 and 0.9812, Sensitivity reached 0.8576, 0.8703 and 0.8493, respectively. In addition, PCAT-UNET also achieved good results in two other F1-Score and Specificity indicators.

## 1 Introduction

Retinal examination can provide important clinical information for the diagnosis of various retinal diseases such as diabetic retinopathy. However, artificial retinal examination requires some clinicians or experts with strong expertise to screen a large number of retinal vessels images. This process is time-consuming, tedious and difficult to implement batch processing, and may lead to diagnostic errors due to subjective factors [1]. In order to alleviate the shortage of medical resources and reduce the workload of doctors and specialists, retinal diseases require a computer-assisted automatic, high-performance retinal vessels segmentation system for pre-screening and other examinations. The automatic retinal vessels segmentation system can quickly and accurately obtain the structural characteristics of retinal vessels, in which branch points and curves can be used to aid the diagnosis and analysis of cardiovessels disease and diabetic retinopathy. The change characteristics of retinal vessels width obtained after segmentation can be used to detect and analyze hypertension [2]. Therefore, the current research on automatic segmentation of retinal blood vessels is an important development direction in this field, and is also of great significance to the study of related retinal diseases [3, 4].

In recent decades, automatic segmentation of retinal vessels has been developing rapidly, and researchers have proposed a large number of retinal vessel segmentation methods. Some traditional methods [5–10] have successfully performed automatic segmentation of retinal vessels images and obtained good segmentation, but they cannot fully characterize the image features, resulting in inadequate detection of retinal structural features and unsatisfactory segmentation accuracy, which still cannot meet the needs of clinical diagnosis for auxiliary ophthalmologists.

Compared with traditional methods, CNN method combines the advantages of medical image segmentation method and semantic segmentation method, making them achieve remarkable performance. Many previous excellent works [4, 11–15] have shown excellent segmentation performance in retinal vessels segmentation, which proves that CNN has strong feature representation learning and recognition ability. But due to the local inherent convolution operation, with the increase of the expansion of training data and the network layer, these methods are hard to learn an explicit global and long-term semantic information interaction [16], the algorithm segmentation results in small blood vessels loss bifurcation and complex discrete curvature form, retinal vessels characteristics to distinguish between edge and background region is not obvious.

Recently, Transformer [17], as an efficient network structure that relies on self-attention to capture global information over long distances, has made remarkable achievements in the field of natural language processing. Many people consider that global information is also needed in visual tasks, and proper Transformer applications can help overcome the limitations of CNN. Therefore, researchers have devoted a great deal of effort to explore suitable Transformer for visual tasks. Early on, literature [18] tried to use CNN [19] to extract deep features, which were then fed into Transformer for processing and regression. Dosovitskiy [20] and Cao [21] both proposed a pure Transformer network to classify and segment images respectively, and achieved great success. They split the image into multiple patches and use each vectorized patch as a word/tag in the NLP so that Transformer can be adopted directly. Subsequently, building on the success of VIT, there has been a large literature of better Transformer based architectures [22–27] that have achieved better performance than CNN. However, the vision transformer still has the problem of a large amount of calculation and insufficient local information extraction.

**Fig 1:**
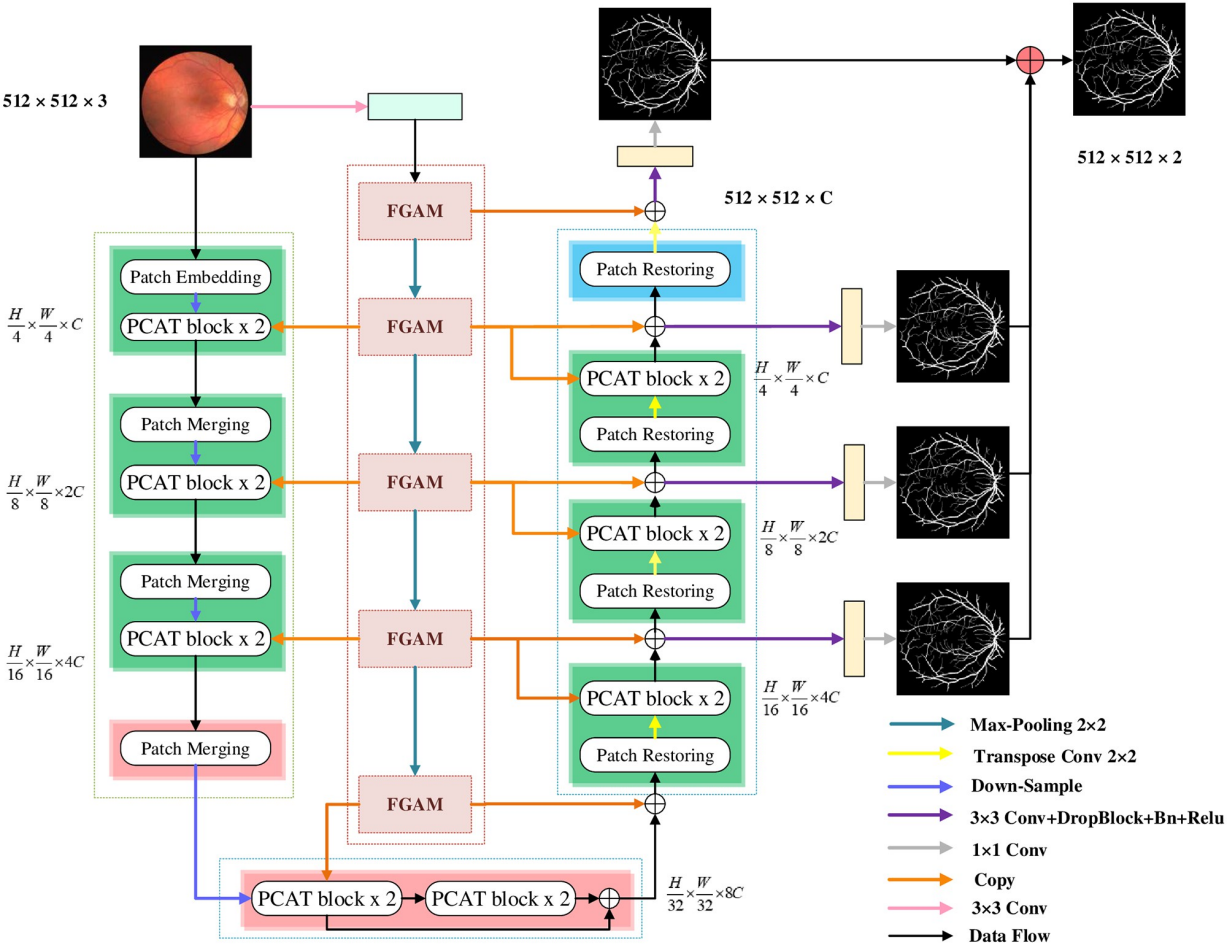
The proposed architecture of PCAT-UNet. Our PCAT-UNet is composed of encoder and decoder constructed by PCAT blocks, convolutional branch constructed FGAM, skip connection and right output layer. In our network, the PCAT block is used as a structurally sensitive skip connection to achieve better information fusion. Finally, the side output layer uses the fused enhanced feature map to predict the vessels segmentation map of each layer along the decoder path.

Aiming at the above problems, we proposed Patches Convolution Attention based Transformer UNet(PCAT-UNet) architecture, especially for 2D retinal vessels image segmentation. Its goal is to combine Transformer and CNN more effectively and perfectly, complementing each other’s shortcomings. As shown in Fig 1, inspired by the success of [21, 28–30], we proposed Patches Convolution Attention Transformer (PCAT) block. It can better combine the advantages of local feature extraction in CNN with the advantages of global information extraction in Transformer. In addition, Feature Grouping Attention Module(FGAM) is proposed to obtain more detailed feature maps of multi-scale characteristic information to supplement the detailed information of retinal vessels. The features obtained are input to encoders and decoders built on both sides based on PCAT for feature fusion to further learn deep feature representation. On this basis, we use PCAT block and FGAM to construct a new U-shaped network architecture with skip connections named PCAT-UNet. A large number of experiments on DRIVE, STARE and CHASE_DB1 datasets show that the proposed method achieves good results, improves the classification sensitivity, and has good segmentation performance. In conclusion, our contribution can be summarized as follows:

- We designed two basic modules, PCAT and FGAM, which are both used to extract refined feature maps with rich multi-scale feature information and to integrate feature information extracted from them to achieve complementary functions.
- On the basis of [29], we improved the self-attention of the original Transformer and proposed the attention between different patches in the feature map and the attention between pixels in a patch. They are Cross Patches Convolution Self-Attention (CPCA) and Inner Patches Convolution Self-Attention (IPCA). This not only reduces the amount of calculation as the input resolution increases, but also effectively maintains the interaction of global information.
- Based on the PCAT block, we constructed a U-shaped architecture composed of encoder and decoder with Skip Connection. The encoder extracts spatial and semantic information of feature images by down-sampling. The decoder up-samples the deep features after fusion to the input resolution and predicts the corresponding pixel-level segmentation. Furthermore, we integrate the convolutional branch based on FGAM module into the middle of the U-shaped structure, and input the feature information extracted from it into the encoder and decoder on both sides to fuse depth and shallow features to improve the learning ability of the network.
- In addition, we added the DropBlock layer after the convolutional layer in the convolutional branch and side output, which can effectively suppress over-fitting phenomenon in training. Experiments show that our model has some performance advantages over the CNN-based retinal vessel segmentation model, which improves its classification sensitivity and has good segmentation comprehensive performance.

## 2 Relate work

### 2.1 Early traditional methods

Among many effective traditional methods proposed, Chaudhuri et al. [3] first proposed a feature extraction operator based on the optical and spatial characteristics of the object to be recognized. Gaussian function was used to encode the vessels image, achieving good segmentation effect, but the structural characteristics of retinal vessels were ignored, and the calculation time was long. Subsequently, Soares et al. [5] used two-dimensional Gabor filter to extract retinal vessels image features, and then used Bayesian classifier to divide pixels into two categories, namely retinal vessels and background. Yan et al. [9] used mathematical morphology to smooth vessels edge information, enhance retinal vessels images and suppress the influence of background information, then applied fuzzy clustering algorithm to segment the enhanced retinal vessels images, and finally purified the fuzzy segmentation results and extracted vessels structures.

### 2.2 Network based on CNN

For the past ten years, convolutional neural network based retinal vessel segmentation methods have been able to predict the pixel points of each vessel and non-vessel category in retinal vessels images, and obtain structural information including the bifurcation pattern, scale size and complex curvature of retinal vessels. Wang et al. [11] used CNN to extract the features of retinal vessels, and then combined with random forest (RF) method to segment the vessels, which improved the segmentation accuracy but required too long training time. In addition, Ronneberger et al. [12] proposed u-NET based on FCN. It uses skip-connection to fuse shallow features extracted from encoder and deep features extracted from decoder, so as to obtain more detailed features and improve the precision of edge segmentation of micro vessels. Di Li et al. [31] adopted a new residual structure and applied attention mechanism to improve segmentation performance in the process of jumping connection. Oliveira et al. [15] proposed a patch-based full-convolutional network segmentation method for retinal vessels. Compared with traditional methods, CNN method combines the advantages of medical image segmentation method and semantic segmentation method, and shows good segmentation performance in retinal vessels datasets, which proves that CNN has strong feature learning and recognition ability. Based on the merits of CNN, we propose a Feature Grouping Attention Module(FGAM), which can extract rich multi-scale feature maps.

### 2.3 Network based on visual transformer

In reference [20, 23, 26, 28, 29, 32, 33], transformer is introduced into visual tasks as an image extraction device. It is helpful to overcome the problem that CNN needs to stack more layers to expand the receptive field by using the multi head self attention mechanism and properly adjusting the transformer. In [20], ViT (Vision Transformer) takes the two-dimensional image block embedded with location information as the input of image recognition task, and has achieved the same performance as the network based on CNN on the large dataset. Literature [23] extends the applicability of transformer and proposes a hierarchical network. Swin Transformer is taken as the backbone of the network structure, and each patch is used as a window to extract the internal correlation of patches, and offset windows are used to capture more features. However, the interaction between local information interaction and adjacent patches in [23] lacks global information interaction. Literature [29] proposed a new attention mechanism in transformer, which captures local information by alternating attention within image blocks instead of the whole image, and applies attention to capture global information between image blocks divided from single-channel feature maps. Performance achieved is comparable to current CNN-based and Transformer-based networks. Therefore, inspired by this, we proposed a PCAT block based on the improvement of CAB (Cross Attention Block) in [29]. It can effectively maintain local and global information interaction while avoiding the huge increase of computation with the increase of input resolution.

### 2.4 Self-attention/transformer combined with CNN

In recent years, [32–36] has been committed to combining CNN and transformer more effectively, trying to introduce self-attention mechanism into CNN, and even using self-attention layer to replace part or all of the popular spatial convolutional layer, so as to break the dominant position of CNN in medical image segmentation. In [37], skip connection with additional attention gate is integrated into U-shaped structure for medical image segmentation, but it is still based on CNN method. In [33, 35, 36], the author combines transformer with CNN to design a powerful encoder for medical image segmentation. At present, most combinations of Transformer and CNN are applied to medical image segmentation such as heart segmentation [32] and multi-modal brain tumor segmentation [38]. Different from the above methods, we propose to integrate the convolutional branch based on CNN into the U-shaped structure based on Transformer to explore the application potential of our model in retinal vessels segmentation to improve the segmentation ability of the model by utilizing the complementarity of Transformer and CNN, and try to explore the application potential of our model in retinal vessels segmentation.

## 3 Method

In this section, we will detail general framework which we proposed for a fusion convolution and transformer like UNet network for retinal vessels segmentation. Specifically, section 3.1 mainly describes the overall network framework proposed by us, as shown in Fig 1. Then, in Sections 3.2 and 3.3, we detail the two main base modules: Patches Convolution Attention Based Transformer(PCAT) block and Feature Grouping Attention Module (FGAM). Finally, in Section 3.4, we describe other important modules of the network model.

### 3.1 Architecture overview

The overall architecture we propose of PCAT-UNet as shown in Fig 1. PCAT -UNet consists of encoder, decoder, convolution branch, skip connection and side output layer. Its basic units are PCAT block and FGAM. Patch embedding module is adopted according to to [23, 29], and 4 × 4 convolution with stride 4 is used to divide it into $\frac{H}{4}\times \frac{W}{4}$ patch tokens without overlap to transform 2D input image into one-dimensional sequence embedding. Each patch was flattened to 48 (4 × 4 × 3) elements, and the feature channel of each token was extended from 48 to C with a linear embedding layer. Then we superimposed several PCAT blocks and patch merging layers for feature extraction at different scales to obtain layered element representations. The patch merging layer implements down-sampling and adding channel dimensions, and the PCAT block completes the learning process of feature representation.

Due to the redundancy of image information in middle convolutional branch, we convolved the input image first and preliminarily extracted the shallow features, which can reduce the memory usage. Then, the convoluted feature map is input into the convolutional branch stacked by five FGAM to extracte local feature information. Because the local feature of convolution can better extract the detailed features of the retinal microvessels, we input them to the encoder and decoder, which can integrate the local feature information of the image with the global feature information learned by transformer module to supplement the detailed features well.

Inspired by U-Net [12], which has a good symmetric structure, we designed a symmetric decoder based on PCAT. The decoder is composed of PCAT blocks and patch restoring layer stacked. Unlike the patch merging layer, the patch restoring layer is specifically designed for up-sampling. In order to reduce the loss of spatial information caused by down-sampling, we propose a skip connection, which fuses the output of the previous module of each patch restoring layer with the output of the middle convolutional branch, and the fusion result is the input of each patch restoring layer. Each patch restoring layer reshapes the input feature maps into large feature maps with a resolution of 2 times. Then, after deep features produced by each layer’s PCAT block are fused with spatial local features from the convolutional branch, up-sampling is performed at the corresponding multiples to restore the resolution of the feature map to the input resolution. Finally, DropBlock, Batch Normalization, and ReLu are performed on the features sampled at each layer. And then through 1 × 1 convolution to output the pixel level segmentation prediction graph. We describe each module in detail below.

### 3.2 Patch convolution attention based transformer block

**Fig 2:**
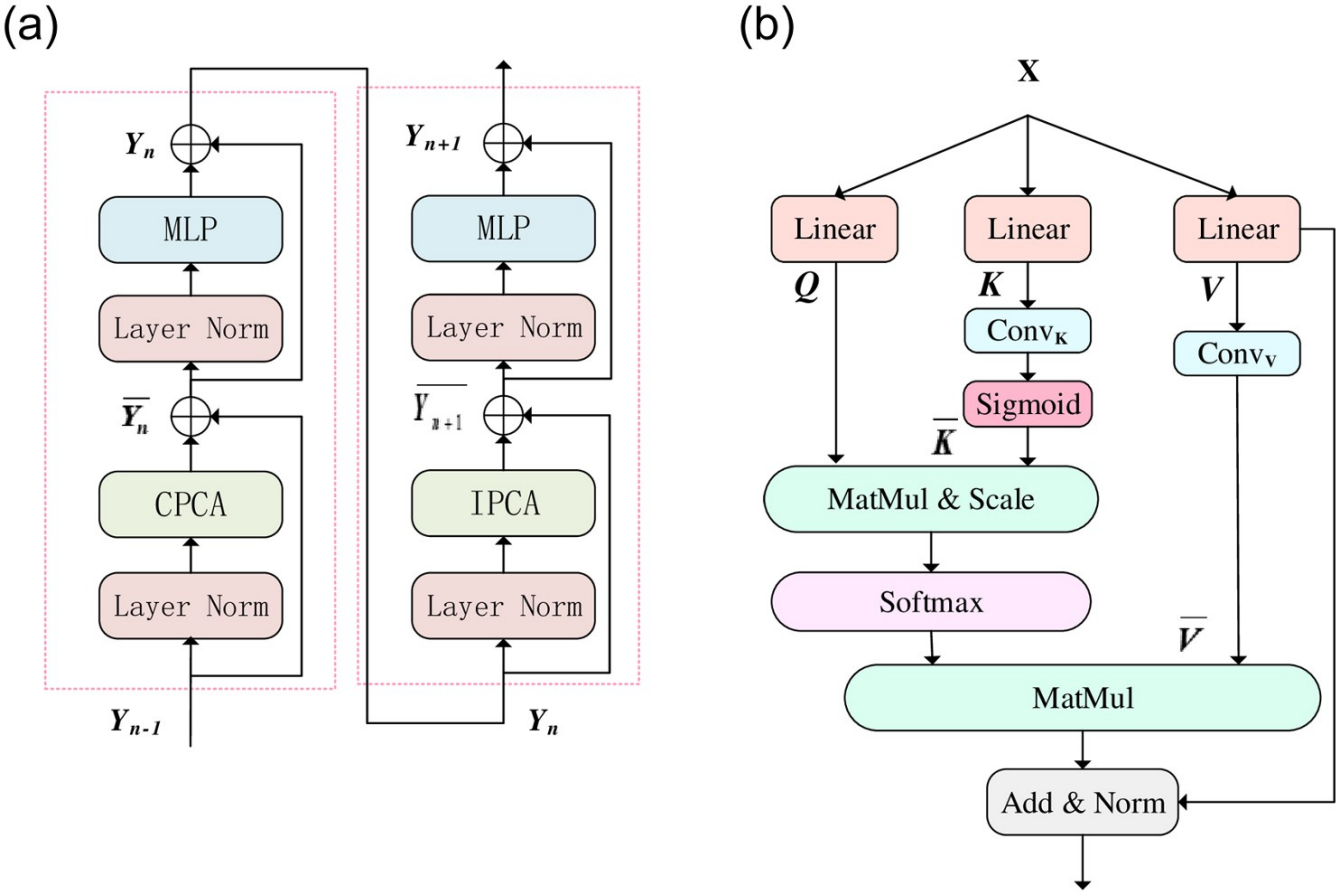
Structure diagram of PCAT block and PCA. (a) two consecutive PCAT blocks.CPCA and IPCA are multi-head self-attention modules with cross and inner patching configurations, respectively. (b)The detailed structure of the PCA, a MHSA based on convolution.

In Fig 2(a), There are two continuous PCAT(Patch Convolution Attention-based Transformer) blocks, in which feature channels and resolution remain unchanged. The traditional transformer is built on the basis of multi-head self-attention (MHSA) [20], which concatenates the results of multiple heads and transforms them using a FeedForward network. Similarly, each PCAT block consists of a LayerNorm (LN) layer, multi-head attention modules, residual links, and MLPs. However, the multi-head Attention modules we have continuously used are Cross Patches Convolution Self-Attention (CPCA) Module and Inner Patches Convolution Self-Attention (IPCA) module. The CPCA module is used to extract the attention between patches in a feature map, while the IPCA module is used to extract and integrate the global feature information between pixels in one patch. Based on such patches partitioning mechanism, continuous PCAT blocks can be expressed as:

$$\begin{array}{cc} & \bar{Y_{n}}=CPCA\left(LN\left(Y_{n-1}\right)\right)+Y_{n-1} \end{array}$$ (1)

$$\begin{array}{cc} & Y_{n}=MLP\left(LN\left(\bar{Y_{n}}\right)\right)+\bar{Y_{n}} \end{array}$$ (2)

$$\begin{array}{cc} & \bar{Y_{n+1}}=IPCA\left(LN\left(Y_{n}\right)\right)+Y_{n} \end{array}$$ (3)

$$\begin{array}{cc} & Y_{n+1}=MLP\left(LN\left(\bar{Y_{n+1}}\right)\right)+\bar{Y_{n+1}} \end{array}$$ (4)

where *Y*_*n*−1_ and *Y*_*n*+1_ are the input and output of the PCAT block respectively. *Y*_*n*_ and *Y*_*n*+1_ are the output of the CPCA module and IPCA module respectively. The size of image patches on the CPCA module and IPCA module is 8×8, and the number of heads is 1 and 8 respectively.

#### 3.2.1 Cross Patches Convolution Self-Attention

The size of the receptive field has a great influence on the segmentation effect for retinal vessel segmentation. Convolutional kernels are often stacked to enlarge the receptive field in models based on convolutional neural networks. In practice, we expect the final receptive field to extend over the entire frame. Transformer [20, 26] is naturally able to capture global information, but not enough local details. As each single-channel feature map naturally has global spatial information, we proposed the Cross Patches Convolution Self-attention (CPCA) module inspired by this. The single-channel feature map is divided into patches, which are stacked and reshaped into original shapes by CPSA module. Patch Convolution self-attention (PCA) in the CPCA module can extract semantic information between patches in each single-channel feature map, and the information exchange between these patches is very important for obtaining global information in the whole feature map. This operation is similar to the depth-separable convolution used in [39, 40], which can reduce the number of parameters and operation cost.

We improved and proposed the CPCA Module based on [29], and expected that the final receptive field in the experiment could include the whole feature map. Specifically, the CPCA Module divides each single-channel feature map into patches of $\frac{H}{N}\times \frac{W}{N}$ in size, and then uses PCA in the CPCA module to obtain different features among patches in each single-channel feature map. Since the number of heads is set to be the same as the patches size, which is not useful for performance, and each head self-attention could have noticed different semantic information among image patches, the number of heads is set to 1 in our experiment.

The multi-head self-attention adopted by CPCA Module is PCA. A little different from the previous work [23, 29, 41], PCA is the MHSA based on convolution, and its detailed structure is shown in Fig 2(b). Specifically, the divided patches $X\in R^{M^{2}\times d}$ are projected into query matrix $Q\in R^{M^{2}\times d}$ key matrix $K\in R^{M^{2}\times d}$ and value matrix $V\in R^{M^{2}\times d}$ through three 1 × 1 linear convolutions, where *M*^2^ and *d* represent the number of patches and dimension of query or key respectively. In the next step, we reshape K and V into 2D space, conduct a 3 × 3 convolution operation and Sigmoid operation successively for the reshaped K, and then expand the result into a one-dimensional sequence to obtain K. Similarly, after 3 × 3 convolution operation, the reshaped V is also expanded into a 1-dimensional sequence to obtain V. Transformer has a global receptive field and can better obtain long-distance dependence, but it lacks in obtaining local detail information. However, we apply a two-dimensional convolution operation on K and V to better supplement local detail feature information. So, the proposed formula for self-attention is now:

$$\begin{array}{c} Attention(Q,K,V)=Softmax\left(\frac{Q\times {\left(\sigma \left(\delta \left(K\right)\right)\right)}^{T}}{\sqrt{d}}\right)\delta \left(V\right)+V \end{array}$$ (5)

$$\begin{array}{c} Attention(Q,K,V)=Softmax\left(\frac{Q\times {\left(\sigma \left(\delta \left(K\right)\right)\right)}^{T}}{\sqrt{d}}+B\right)\delta \left(V\right)+V \end{array}$$ (6)

where $Q,K,V\in R^{M^{2}\times d}$ are query, key and value matrices, *M*^2^ and *d* are patch number and query or key dimension respectively. $B\in R^{M^{2}\times M^{2}}$ whose value is taken from the deviation matrix ∈ *R*^(2*M*−1)×(2*M*+1)^, *δ* represents a 3 × 3 convolution operation on the input, *σ* represents the sigmoid function, and then expands the result into a one-dimensional sequence, so *K* = *σ*(*δ*(*K*)), *V* = (*δ*(*V*). The acquired attention is added with a residual connection V to supplement the information lost by convolution, and the final self-attention is obtained.

#### 3.2.2 Inner Patches Convolution Self-Attention

Our PCAT block is designed to combine attention between patches with attention within patches. Different semantic information between patches can be obtained through CPCA to realize the information exchange of the whole feature map. But the interrelationship between pixels within patches is also crucial. The proposed Inner Patches Convolution self-attention (IPCA) module considers the relationship between pixels within Patches and can obtain the Attention between pixels within each patch.

Inspired by the local feature extraction characteristics of CNN, we introduce the locality of convolution method in CNN into Transformer. IPCA Module divides multiple-channel feature images into patches with a size of $\frac{H}{N}\times \frac{W}{N}$, regards each patch divided as an independent attention range, and uses multi-head self-attention for self-attention of all pixels in each patch instead of the whole feature map. Multi-head self-attention used in IPCA module is also PCA. However, CPCA module extracts self-attention from a complete single-channel feature map, while IPCA Module is self-attentional from pixels in each patch of multi-channel feature maps. In addition, PCA of the two are slightly different. The self-attention in IPCA Module adopts relative position encoding, so its self-attention calculation is shown in Eq (6).

### 3.3 Feature Grouping Attention Module

**Fig 3:**
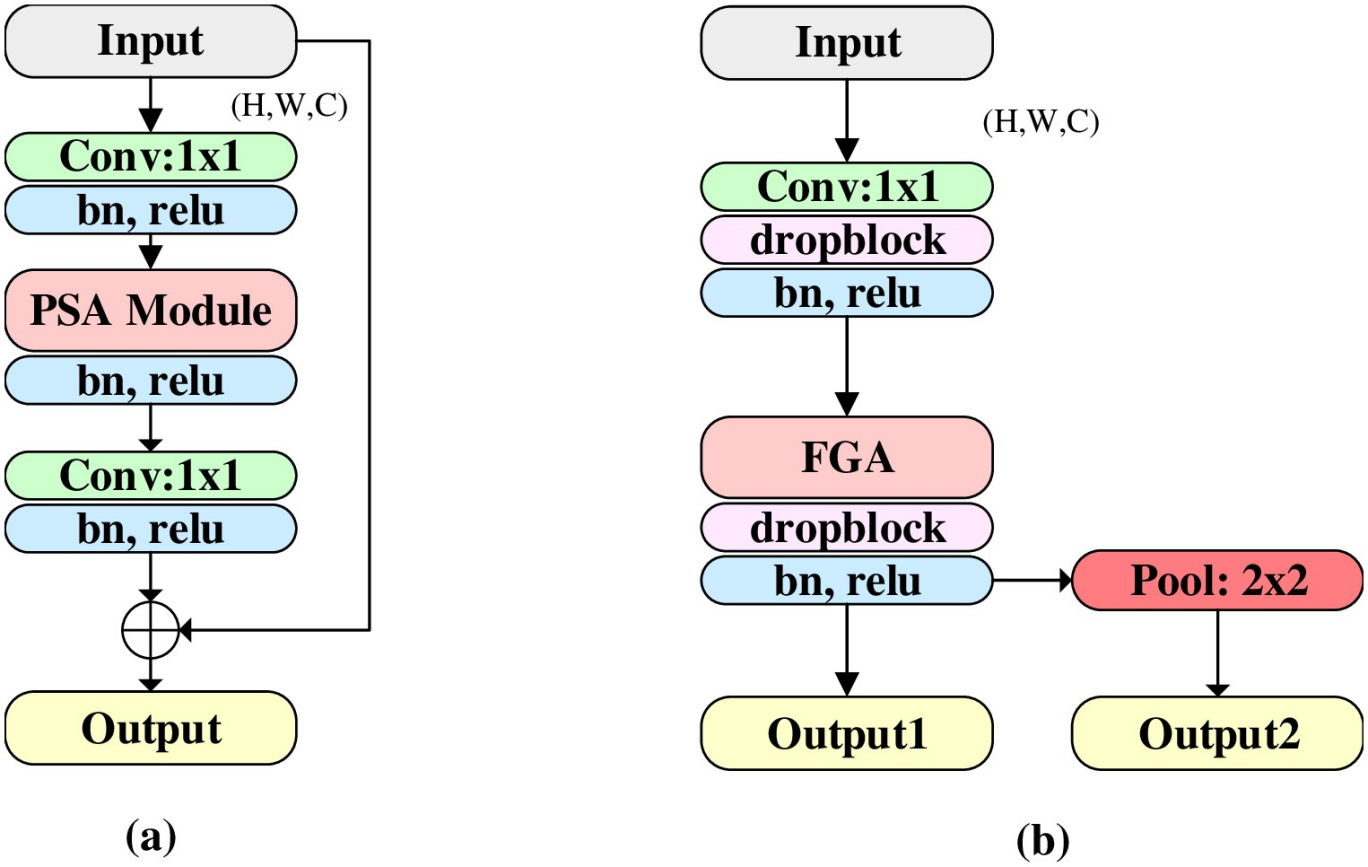
Structure comparison of EPSA module and FGAM. (a) EPSA module; (b) FGAM.

In [30], a new pyramid segmentation attention (EPSA) module is used to build an efficient backbone network. The structure of EPSA module is shown in Fig 3(a), which has strong multi-scale representation ability and can adaptively re-calibrate the weight of cross-dimensional channels. However, for the images of retinal blood vessels processed by us, the large-scale convolution adopted by PSA in EPSA module is easy to cause the loss of features of tiny blood vessels, thus failing to obtain good segmentation results of marginal blood vessels. On this basis, we propose an effective Feature Grouping Attention Module(FGAM), as shown in Fig 3(b). Different from EPSA module, FGAM only adopts a 1×1 convolution, replacing the original final convolution layer with a 2×2 pooling layer to realize the function of sampling under the convolution branch, and adding a DropBlock [42] after convolution and FGA. DropBlock combined with Batch Normalization layer (BN) and ReLu activation unit, can effectively prevent network overfitting and speed up network convergence.

In addition, in order to reduce the loss of vessel edge features, FGA(feature grouping attention) in FGAM does not employ the original pyramid structure (e.g. group convolution kernels are 3, 5, 7, 9), but small scale grouping convolution is used (group convolution kernels are 1, 1, 3, 3), and different multi-group convolution kernels are used for parallel processing to obtain channel feature maps with different spatial resolutions. Specifically, our FGA divides the channels of input feature map X into part S(default is 4) on average, and applies multiple groups of convolution (default group size of each part is 2,4,8,8 respectively) to the corresponding part to extract small-scale features, so as to obtain different feature maps on the channel. Then, SEWeight module [43] was used to extract the attention of feature images in different proportions, and Softmax was used to calibrate the channel attention vector. Finally, the re-calibrated weight and corresponding feature maps were used for element-level product operation. Therefore, using FGA, we can integrate multi-scale spatial information and cross-channel attention into each feature grouping block, obtain better information fusion between local and global channel attention, and obtain feature maps with richer details. This process follows the method of [30] and will not be described in this paper.

In order to verify the segmentation performance of FGAM in the proposed model on retinal vessels, we replaced FGAM in the proposed method PCAT-UNet with EPSA module in Sections 4.2.2 ablation experiment to obtain the PCAT-UNet (EPSA) model. Then, its segmentation performance on DRIVE, STARE and CHASE_DB datasets was compared with that of the proposed method in this paper, as shown in Table 9. Finally, we will analyze the comparison results in Sections 4.4.2 below.

### 3.4 Other important modules

#### 3.4.1 Patch embedding layer

Our patch embedding layer consists of an up-sampling and linear projection layer (which is a 4 × 4 convolution with stride 4.). For retinal vessels images, the larger the pixel, the better the segmentation effect. Therefore, the 512 × 512 input image was up-sampling twice, and the image was enlarged to 1024 × 1024 pixels. Then it is divided into non-overlapping $\frac{H}{4}\times \frac{W}{4}$(H = 1024, W = 1024) patches of tokens, and each fragment is flattened into 48 elements. Finally, the feature dimension of each token is extended from 48 to C with a linear embedding layer.

#### 3.4.2 Patch merging layer

In the encoder based on PCAT module, the patch merging layer is used to reduce the number of tokens and increase the feature dimension. Specifically, patches are divided into four parts, which can reduce the resolution of features by a factor of 2 and increase the dimension of features by a factor of 4 by splicing. Feature information is easily lost during down-sampling, so we consider using FGAM to obtain better information fusion between local and global channel attention to supplement detail features. FGAM here maintains the feature dimension and resolution unchanged. Finally, a linear layer is applied to the fusion feature, turning the feature dimension into twice the input dimension. This process is repeated three times in the encoder.

#### 3.4.3 Patch restoring layer

Corresponding to the encoder is a symmetric decoder based on the PCAT module. Patch restoring layer is used in the decoder to up-sample deep features and reduce their feature dimensions. Like the patch merging layer, patch restoring layer also uses FGAM to obtain better information fusion between local and global channel attention, and then adopts deconvolution operation to reshape the input into a feature map with higher resolution (2× up-sampling), and then reduces its feature dimension to $\frac{1}{2}$ of the input dimension. This process is repeated four times in the decoder.

#### 3.4.4 DropBlock

DropBlock [42] is a structured form of dropout that can effectively prevent network overfitting problems. In the feature map, it discards contiguous regions rather than individual random units. In this way, part of redundant semantic information can be effectively discarded and more effective information can be encouraged to be learned on the network. We use a DropBlock, a Batch Normalization (BN) layer, and a ReLu activation unit to construct a structured module. This module is used in both the side output layer in our network and in FGAM. See Figs 1 and 3(b).

## 4 Experiment

In this chapter, we describe model experiments to evaluate our architecture for retinal vessel segmentation tasks in detail. We describe the datasets and implementation details for the experiment in Sections 4.1 and Sections 4.2, respectively. Then, the evaluation criteria are introduced in Sections 4.3. Finally, Sections 4.4 introduces experimental results and analysis, including comparison with existing advanced methods and ablation experiments to prove the effectiveness of our network.

### 4.1 Datasets

We used three publicly available datasets: DRIVE [44], STARE [45] and CHASE_DB1 [46] to conduct retinal vessels segmentation experiments to evaluate the performance of our approach. Detailed information about these three datasets is shown in Table 1.

**Table 1:** The specific information of DRIVE, STARE and CHASE_DB1 datasets.

Datasets	Quantity	Resolution	Train-Test split
DRIVE	40	565 × 584	Official train-test split
STARE	20	605 × 700	First 16 for train, last 4 for test
CHASE_DB1	28	999 × 960	First 20 for train, last 8 for test

All three datasets have different image formats, sizes and numbers. The DRIVE dataset contains 40 color retinal vessels images in tif format (565 × 584). The dataset consists of 20 training images and 20 test images. The STARE dataset consisted of 10 with and 10 without lesions color retinal vessels images in a PPM (605 × 700) format. The CHASE_DB1 dataset contains 28 images in JPG (999 × 960) format. Unlike the DRIVE dataset, the STARE and CHASE_DB1 datasets do not have a formal classification of training and test datasets. To better compare with other methods, we follow the same protocol for data segmentation as [47]. For the STARE dataset, 16 images were selected as the training set and the remaining 4 images as the test set. In the CHASE_DB1 dataset, 20 images were selected as the training set and the remaining 8 images as the test set. In addition, the DRIVE dataset comes with a FoV mask, while the other two datasets do not provide FoV masks. For fair evaluation, we use FoV masks for the two datasets provided by [47]. Finally, each dataset test set contains two sets of comments, and in keeping with the other methods, we used the comments from the first group as ground truth to evaluate our model.

**Fig 4:**
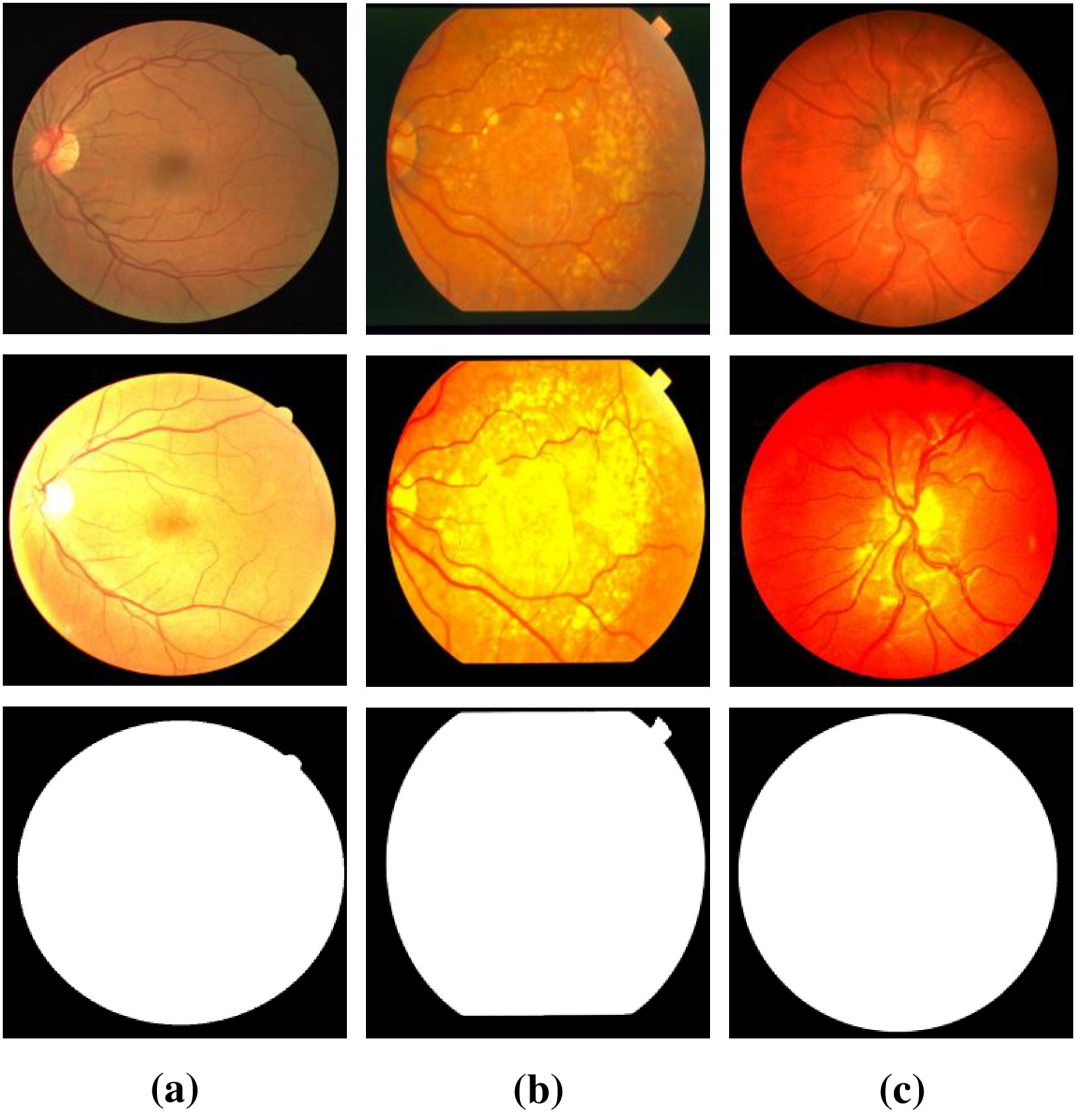
Retinal vessels images of the dataset (line 1), corresponding retinal vessels images generated by histogram stretching enhancement (line 2) and corresponding FOV mask (line 3); (a) DRIVE (b) STARE (c) CHASE_DB1.

In Table 1, we noticed that the original size of the three datasets was not suitable for the input image size of our network, so we adjusted the image size of the datasets and unified it into 512×512 image as the input. In addition, to increase the number of training samples, we adopted the histogram stretching data enhancement method for all three datasets, doubling the training sets of the three original datasets to 40, 32 and 40 images respectively. In Fig 4, retinal vessels images of these three datasets, corresponding retinal vessels images generated by histogram stretching enhancement method and corresponding FOV mask can be seen.

### 4.2 Implementation details

#### 4.2.1 Settings

In the experiment of retinal vessel segmentation, we train the network for 250 epochs. The initial learning rate is 0.001 and decays 10 times every 50 epochs. And we set the batch size to 4. Adam optimizer and binary cross entropy loss function were used to train the PCAT-UNet network. The entire network trains from scratch, with no additional training data. We trained the network on DRIVE, STARE and CHASE_DB1 training sets and evaluated it on the respective validation sets for each dataset. Our experiment was conducted on an NVIDIA Tesla V100 32GB GPU.

#### 4.2.2 Implementation

The DropBlock module is used in both FGAM and the side output layer in our network. DropBlock takes two main parameters: block_size, which specifies the size of the block to be discarded, and *γ*, which specifies the number of features to be discarded. The DropBlock parameters in FGAM are set to block_size = 7, *γ* = 0.9, and the DropBlock parameters in the side output layer are set to block_size = 5, *γ* = 0.75. The input and output sizes of the network are 512 x 512.

#### 4.2.3 Data augmentation

The input of our network is the image of the entire retinal blood vessels, and the output is consistent with the input size. And we observe that data augmentation can overcome the problem of network overfitting, so we use some data augmentation methods to improve the network performance. The augmentation methods used in the network include gaussian transform with probability 0.5, where Sigma = (0, 0.5), random rotation in the range [0,20°], random horizontal flip with probability 0.5, and gamma contrast enhancement in the range [0.5, 2]. These methods can enhance the training image and avoid network overfitting, which can greatly improve the performance of the model.

### 4.3 Evaluation criteria

The process of retinal blood vessel segmentation is the classification of pixels. All pixels are classified as vessel pixels or background pixels. To evaluate the performance of our model, we compared the segmentation results with the corresponding ground truth and divided the comparison results of each pixel into true positive (TP), false positive (FP), false negative (FN) and true negative (TN). Then, we adopted specificity (Sp), sensitivity (Se), accuracy (ACC), F1-Score (F1) and AUC: area under the receiver operating characteristic (ROC) as the measurement indexes:

$$\begin{array}{cc} & Sp=\frac{TN}{TN+FP} \end{array}$$ (7)

$$\begin{array}{cc} & Se=Recall=\frac{TP}{TP+FN} \end{array}$$ (8)

$$\begin{array}{cc} & Acc=\frac{TP+TN}{TP+FN+TN+FP} \end{array}$$ (9)

$$\begin{array}{cc} & Precision=\frac{TP}{TP+FP} \end{array}$$ (10)

$$\begin{array}{cc} & F1=2\times \frac{Precision\times Recall}{Precision+Recall}=\frac{2TP}{2TP+FP+FN} \end{array}$$ (11)

where, TP represents positive truth value when a vessel pixel in ground truth is correctly classified in the predicted image, TN represents negative truth value when a non-vessel pixel in ground truth is correctly classified in the predicted image, FN misclassifies the vessel pixels in ground truth as non-vessel pixels in the predicted image, and FP means that the non-vessel pixels in the ground truth are wrongly marked as the vessel pixels in the predicted image. Precision and Recall mean Precision Ratio and Recall Ratio respectively, while the ROC curve represents the proportion of vessels correctly classified as vessel pixels versus misclassified as non-vessel pixels. AUC refers to the area under the ROC curve, which can be used to measure the segmentation performance. The closer the AUC value is to 1, the closer the system performance is to perfection.

### 4.4 Experimental results and analysis

#### 4.4.1 Compare with existing methods

We compared PCAT-UNet architecture with the best existing retinal vessel segmentation methods on DRIVE, STARE and CHASE_DB1 datasets, including UNet [12], DFUNet [1], LadderNet [48], Denseblock-UNet [49], DEUNet [50], IterNet [24], DGUNet [51] and Pyramid U-Net [52], etc. We summarize the release years of these methods and the performance comparisons across the three datasets in Tables 2–4. All three tables report traditional measures such as F1-score, Sensitivity, Specificity, Accuracy and AUC-ROC. For these three datasets, we only count pixels in the FOV.

**Table 2:** Performance comparison of different segmentation methods on the DRIVE dataset.

Methods	Year	F1-score	SE	SP	ACC	AUC
Human Observer	-	N.A	0.7760	0.9724	0.9472	0.8742
U-Net [12]	2015	0.8142	0.7537	0.9820	0.9531	0.9755
Residual UNet [53]	2018	0.8149	0.7726	0.9820	0.9553	0.9779
R2UNet [53]	2018	0.8171	0.7792	0.9813	0.9556	0.9784
DFUNet [1]	2019	0.8190	0.7863	0.9805	0.9558	0.9778
LadderNet [48]	2019	0.8202	0.7856	0.9810	0.9561	0.9793
DEUNet [50]	2019	**0.8270**	0.7940	0.9816	0.9567	0.9772
IterNet [47]	2020	0.8205	0.7735	0.9838	0.9573	0.9816
DGUNet [51]	2020	N.A	0.7614	0.9837	0.9604	0.9846
Nest U-Net [54]	2021	0.7863	0.8060	0.9869	0.9512	0.9748
Pyramid U-Net [52]	2021	N.A	0.8213	0.9807	0.9615	0.9815
**PCAT-UNet(Ours)**	**2021**	0.8160	**0.8576**	**0.9932**	**0.9622**	**0.9872**

**Table 3:** Performance comparison of different segmentation methods on the STARE dataset.

Methods	Year	F1-score	SE	SP	ACC	AUC
Human Observer	-	N.A	0.8952	0.9384	0.9349	0.9898
U-Net [12]	2015	0.8373	0.8270	0.9842	0.9690	0.9830
DenseBlock-UNet [49]	2018	0.7691	0.6807	0.9916	0.9651	0.9755
DFUNet [1]	2019	0.7629	0.6810	0.9903	0.9639	0.9758
IterNet [47]	2020	0.8146	0.7715	0.9886	0.9701	0.9881
Nest U-Net [54]	2021	0.8230	0.8230	**0.9945**	0.9641	0.9620
**PCAT-UNet(Ours)**	**2021**	**0.8836**	**0.8703**	0.9937	**0.9796**	**0.9953**

**Table 4:** Performance comparison of different segmentation methods on the CHASE_DB1 dataset.

Methods	Year	F1-score	SE	SP	ACC	AUC
Human Observer	-	N.A	0.7686	0.9779	0.9560	0.8733
U-Net [12]	2015	0.7783	0.8288	0.9701	0.9578	0.9772
DenseBlock-UNet [49]	2018	0.8006	0.8178	0.9775	0.9631	0.9826
DFUNet [1]	2019	0.8001	0.7859	0.9822	0.9644	0.9834
LadderNet [48]	2019	0.8031	0.7978	0.9818	0.9656	0.9839
DEUNet [50]	2019	0.8037	0.8074	0.9821	0.9661	0.9812
IterNet [47]	2020	0.8073	0.7970	0.9823	0.9655	0.9851
DGUNet [51]	2020	N.A	0.7993	0.9868	0.9783	0.9869
Pyramid U-Net [52]	2021	N.A	0.8035	0.9787	0.9639	0.9832
**PCAT-UNet(Ours)**	**2021**	**0.8273**	**0.8493**	**0.9966**	**0.9812**	**0.9925**

**Fig 5:**
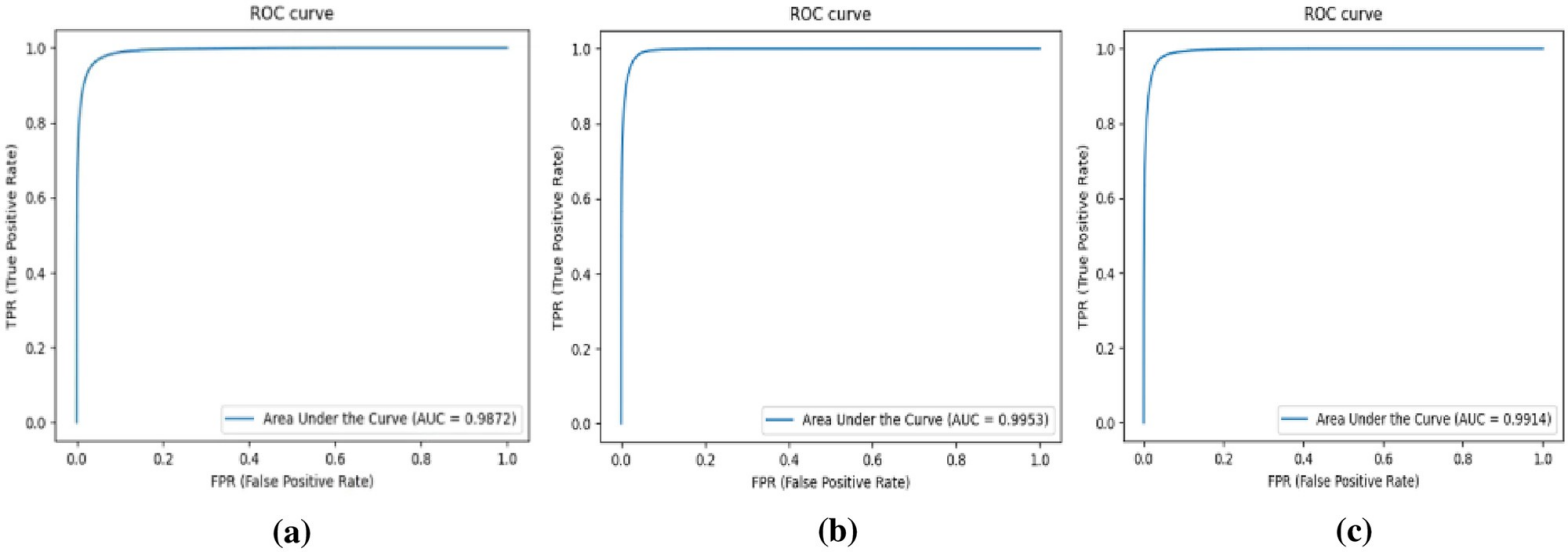
ROC Curves on (a) DRIVE (b) STARE (c) CHASE_DB1.

As shown in Tables 2–4, our model achieves the best performance on DRIVE, STARE, and CHASE_DB1 datasets, significantly outperforming UNet-derived network architectures. Among them, the sensitivity, accuracy and AUC-ROC values (three main indicators of this task) obtained by method we proposed were the highest in the three datasets, which were 0.8576/0.8703/0.8483, 0.9622/0.9796/0.9812, 0.9872/0.9953/0.9925 respectively. In addition, our model achieved the highest F1-score on STARE and CHASE_DB1 datasets (0.8836 and 0.8273, respectively), and the highest specificity on DRIVE and CHASE_DB1 datasets (0.9932 and 0.9966, respectively). Excellent sensitivity indicated a higher True Positive Rate of our method compared to other methods including second-person observers, and excellent specificity indicated a lower False Positive Rate of our method than other methods. This further indicates that, compared with other methods, our proposed method can identify more True Positive and True Negative retinal vessel pixels. In Fig 5, we can see ROC Curves for the three datasets and the high and low True Positive Rates (TPR) and False Positive Rates shown in the figures. Therefore, according to the above analysis results, PCAT-UNet architecture we proposed achieves the most advanced performance in the retinal vessel segmentation challenge.

**Fig 6:**
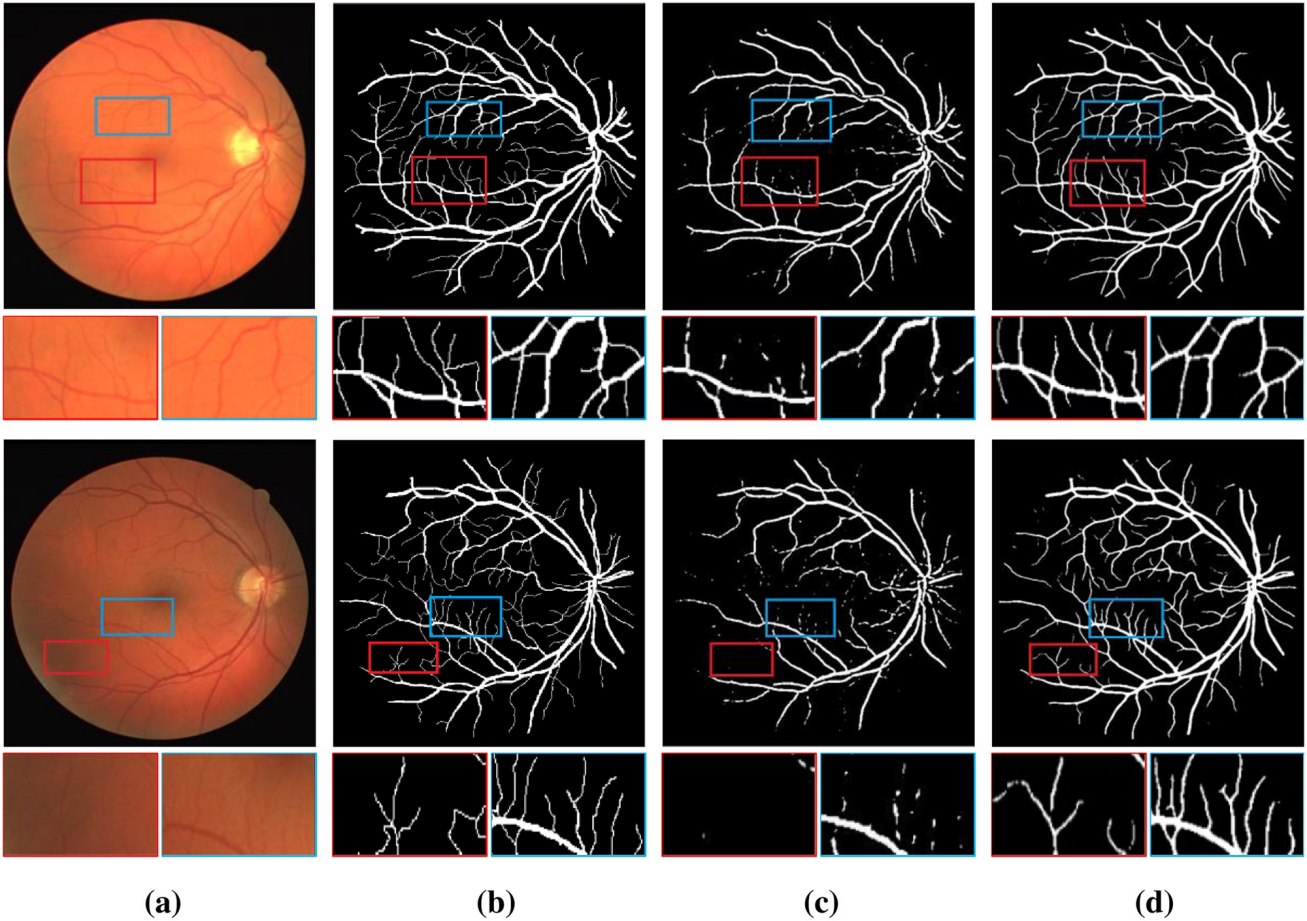
Comparison of example vessel segmentation results on DRIVE dataset: (a) original retinal images; (b) ground truths; (c) segmentation results for UNet; (d) segmentation results for PCAT-UNet. The first row of the image is the whole image, and the second row is the zoomed in area of the marked red border and blue border in the image.

Table 2 shows that compared with the maximum value of each indicator in the existing method on the DRIVE dataset, the PCAT-UNet proposed by us achieved higher performance, in which SE increased by 3.63%, SP by 0.63%, ACC by 0.07% and AUC by 0.26%. F1-score also scored well, with a difference of just 1.1%. We compared the prediction results of the method we proposed with UNet method on DRIVE dataset, as shown in Fig 6. The enlarged details of some specific blood vessels are given in the figure. Some unsegmented microvessels and important cross blood vessels in the segmentation map of UNet can be seen in the segmentation map of the method in this paper. Therefore, the segmentation figure of the method in this paper is more accurate and closer to ground truths than that of the method in UNet.

**Fig 7:**
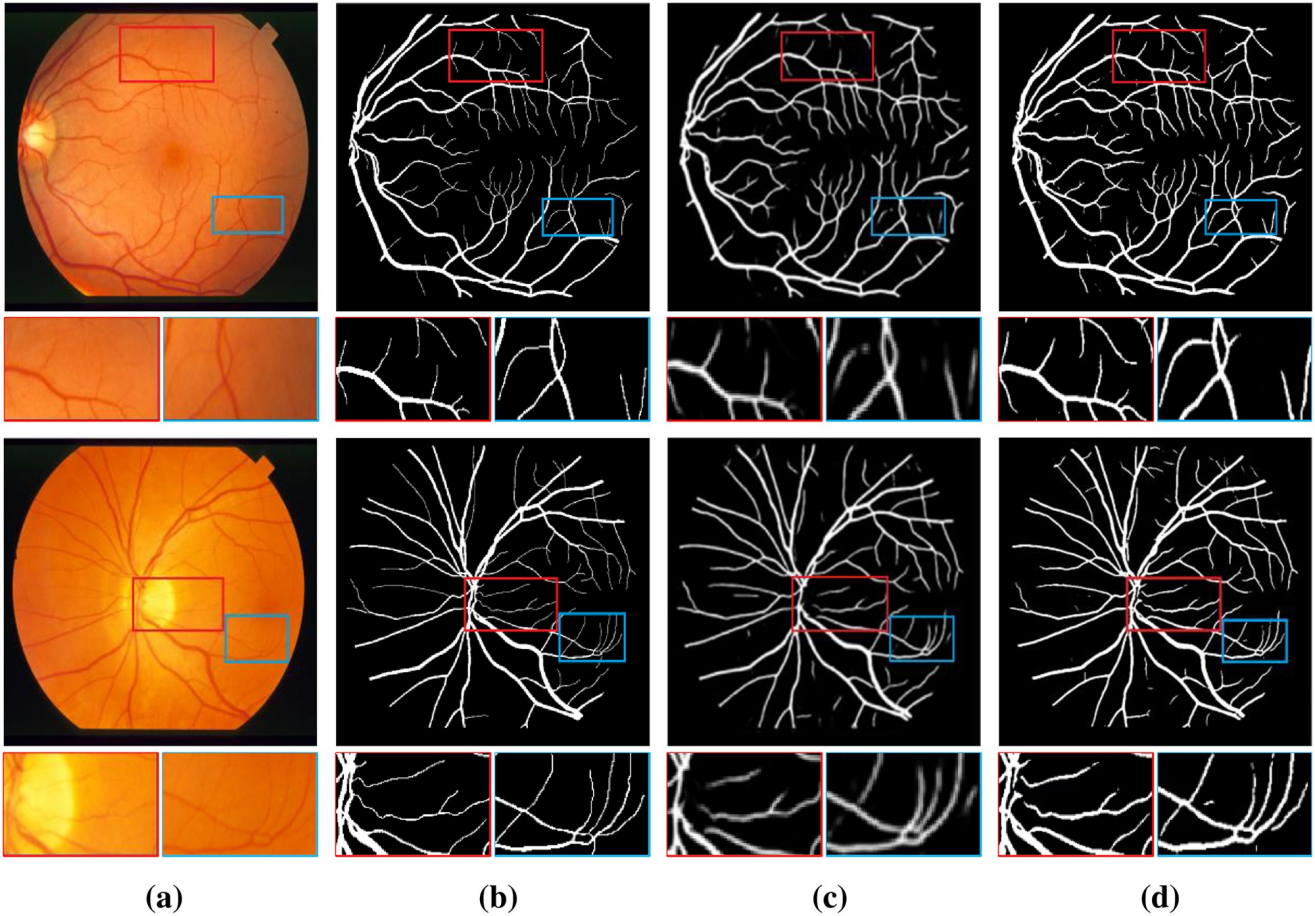
Comparison of example vessel segmentation results on STARE dataset: (a) original retinal images; (b) ground truths; (c) segmentation results for UNet; (d) segmentation results for PCAT-UNet. The first row of the image is the whole image, and the second row is the zoomed in area of the marked red border and blue border in the image.

As can be seen from Table 3, compared with the maximum values of each indicator of existing methods, the method in this paper achieved higher performance in STARE dataset, in which F1-Score, SE, ACC and AUC increased by 4.63%, 4.33%, 0.95% and 0.72% respectively. Moreover, the results of SP are very competitive, with a difference of only 0.08%. In addition, Fig 7 also demonstrates that our approach is more efficient. We can see Fig 7 showing the segmentation results of original retinal images, ground truths, UNet method, and our method on the STARE dataset. It can be observed from the enlarged image that the proposed method has a stronger ability to detect the pixels of cross vessels and can effectively identify the pixels of retinal vessels. The segmentation result is more accurate than that of UNet method.

**Fig 8:**
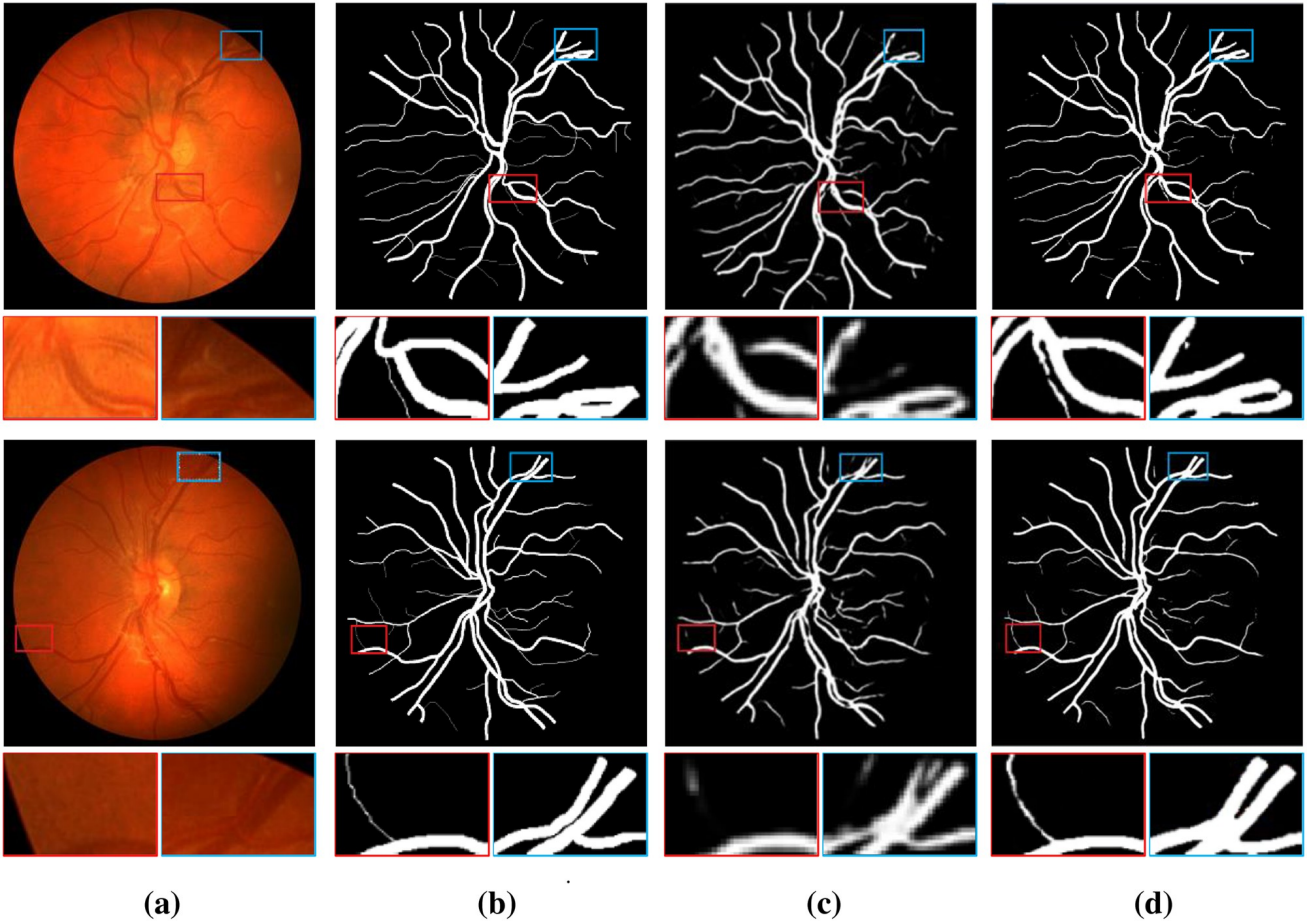
Comparison of example vessel segmentation results on CHASE_DB1 dataset: (a) original retinal images; (b) ground truths; (c) segmentation results for UNet; (d) segmentation results for PCAT-UNet. The first row of the image is the whole image, and the second row is the zoomed in area of the marked red border and blue border in the image.

As can be seen from Table 4, our proposed PCAT-UNet method is superior to the most advanced method in all indicators of the CHASE_DB1 dataset. Among them, compared with the maximum value of each indicator in the existing methods in the table, our experimental results increased F1-Score by 2%, SE by 2.05%, SP by 0.98%, ACC by 0.29% and AUC by 0.56%. Fig 8 also provides a comparison of the segmentation results of original Retinal images, ground truths, UNet method and this method on the CHASE_DB1 dataset. From the enlarged picture of details, we can see that the segmentation effect of the proposed method is better than that of UNet method in the segmentation of micro-vessels, blood vessel intersection and blood vessel edge, which further proves the superiority of our method.

#### 4.4.2 Ablation study

To investigate the effects of different factors on model performance, we conducted extensive ablation studies on DRIVE, STARE and CHASE_DB1 datasets. Specifically, we explore the influence of the backbone, FGAM, convolutional branch and Dropblock on our model. The research results are shown in Tables 5–7. **Backbone**. In the ablation experiment of this paper, we will build a backbone with the pure Transformer module. Specifically, backbone is a U-shaped network composed of encoder constructed by PCAT Block and Patch Embedding Layer and decoder constructed by PCAT Block and Patch Restoring Layer.

**Table 5:** Ablation study results on DRIVE dataset.

Network	F1-score	SE	SP	ACC	AUC
Backbone	0.8043	0.7719	0.9885	0.9606	0.9854
Backbone+FGAM	0.8092	0.7791	0.9909	0.9612	0.9862
Backbone+FGAM+Conv-Branch	0.8118	0.7763	0.9899	0.9615	0.9865
PCAT-UNet	0.8160	0.8576	0.9932	0.9622	0.9872

**Table 6:** Ablation study results on STARE dataset.

Network	F1-score	SE	SP	ACC	AUC
Backbone	0.8497	0.8518	0.9878	0.9750	0.9936
Backbone+FGAM	0.8578	0.8750	0.9911	0.9766	0.9944
Backbone+FGAM+Conv-Branch	0.8597	0.8650	0.9901	0.9770	0.9948
PCAT-UNet	0.8836	0.8703	0.9937	0.9796	0.9953

**Table 7:** Ablation study results on CHASE_DB1 dataset.

Network	F1-score	SE	SP	ACC	AUC
Backbone	0.8104	0.8149	0.9932	0.9797	0.9906
Backbone+FGAM	0.8188	0.8269	0.9966	0.9806	0.9919
Backbone+FGAM+Conv-Branch	0.8193	0.8174	0.9979	0.9809	0.9922
PCAT-UNet	0.8273	0.8493	0.9967	0.9812	0.9925

In these three tables, the first line is the experimental result of backbone built with pure transformer, and the second line is the backbone with FGAM (in Patch Embedding Layer and Patch Restoring Layer). The third line is the backbone with FGAM (in Patch Embedding Layer and Patch Restoring Layer) and convolution branch. The last line results in backbone with FGAM (in Patch Embedding Layer and Patch Restoring Layer), convolution branch and DropBlock, that is, PCAT-UNet proposed by us. As can be seen from the three tables, the network constructed by pure Transformer segmented retinal vessels and achieved good results, but the network combined with Transformer and convolution achieved better segmentation performance. In addition, the introduction of DropBlock also plays an important role in the segmentation results. This proves the effectiveness of these modules for our network.

In terms of time consumption, we compare PCAT-UNET with the Backbone, Backbone+FGAM and Backbone+FGAM+Conv-Branch methods of the proposed model. In the experiment of this paper, all the above algorithms are implemented with Pytorch, and 250 iterations of DRIVE dataset (including 20 original training graphs and 20 data-enhanced graphs) are tested on NVIDIA Tesla V100 32GB GPU. The number of parameters and running time are shown in Table 8.

**Table 8:** Quantitative comparison of parameter and time consumption.

Network	Params(G)	Train time(s)	Test time(s/image)
Backbone	40.1	4875	0.1103
Backbone+FGAM	55.9	9700	0.1302
Backbone+FGAM+Conv-Branch	57.4	12800	0.1584
PCAT-UNet	57.4	12550	0.1547

As can be seen from Table 9, PCAT-UNet (EPSA) method (using EPSA module to replace FGAM in PCAT-UNet method proposed in this paper) also achieved good results in retinal vessel segmentation. Its accuracy, specificity and AUC value were close to PCAT-UNET (FGAM) method, but its sensitivity was lower. It indicates that this method has information loss in vessel feature extraction and needs further improvement. However, the PCAT-UNet (FGAM) method proposed in this paper significantly improved the sensitivity index, indicating that the small-scale grouping convolution in FGAM can fully extract and completely recover the vessel edge information. FGA also focuses on the blood vessels, reducing the influence of background noise region on the segmentation results. It has certain rationality and validity on algorithm level.

**Table 9:** Performance comparison of EPSA module and FGAM on three datasets.

Datasets	Method	F1-score	SE	SP	ACC	AUC
DRIVE	PCAT-UNet(EPSA)	0.8146	0.7990	0.9872	0.9617	0.9867
	PCAT-UNet(FGAM)	0.8160	0.8576	0.9932	0.9622	0.9872
STARE	PCAT-UNet(EPSA)	0.8578	0.8610	0.9924	0.9770	0.9945
	PCAT-UNet(FGAM)	0.8836	0.8703	0.9937	0.9796	0.9953
CHASE_DB1	PCAT-UNet(EPSA)	0.8270	0.8375	0.9966	0.9809	0.9927
	PCAT-UNet(FGAM)	0.8273	0.8493	0.9967	0.9812	0.9925

## 5 Conclusion

In this paper, we propose a U-shaped network with convolution branching based on Transformer. In this network, the multi-scale feature information obtained in the convolutional branch is transmitted to the encoder and decoder on both sides, which can supplement the spatial information loss caused by the down-sampling operation. In this way, local features obtained in CNN can be better integrated with global information obtained in Transformer, so as to obtain more detailed feature information of vessels. Our method achieves the most advanced segmentation performance on DRIVE, STARE and CHASE_DB1 datasets, and greatly improves the extraction of small blood vessels, providing a powerful help for clinical diagnosis. Our long-term goal is to take the best of both Transformer and CNN and combine them more effectively and perfectly, and our proposed PCAT-UNET approach is a meaningful step toward achieving this goal.
